# Identification of a Small TAF Complex and Its Role in the Assembly of TAF-Containing Complexes

**DOI:** 10.1371/journal.pone.0000316

**Published:** 2007-03-21

**Authors:** Màté A. Demény, Evi Soutoglou, Zita Nagy, Elisabeth Scheer, Àgnes Jànoshàzi, Magalie Richardot, Manuela Argentini, Pascal Kessler, Laszlo Tora

**Affiliations:** Institut de Génétique et de Biologie Moléculaire et Cellulaire (IGBMC), Centre National de la Recherche Scientifique (CNRS) UMR 7104, Institut National de la Santé et de la Recherche Médicale (INSERM)U 596, Université Louis Pasteur de Strasbourg, Illkirch, Strasbourg, France; University of Hong Kong, China

## Abstract

TFIID plays a role in nucleating RNA polymerase II preinitiation complex assembly on protein-coding genes. TFIID is a multisubunit complex comprised of the TATA box binding protein (TBP) and 14 TBP-associated factors (TAFs). Another class of multiprotein transcriptional regulatory complexes having histone acetyl transferase (HAT) activity, and containing TAFs, includes TFTC, STAGA and the PCAF/GCN5 complex. Looking for as yet undiscovered subunits by a proteomic approach, we had identified TAF8 and SPT7L in human TFTC preparations. Subsequently, however, we demonstrated that TAF8 was not a stable component of TFTC, but that it is present in a small TAF complex (SMAT), containing TAF8, TAF10 and SPT7L, that co-purified with TFTC. Thus, TAF8 is a subunit of both TFIID and SMAT. The latter has to be involved in a pathway of complex formation distinct from the other known TAF complexes, since these three histone fold (HF)-containing proteins (TAF8, TAF10 and SPT7L) can never be found together either in TFIID or in STAGA/TFTC HAT complexes. Here we show that TAF8 is absolutely necessary for the integration of TAF10 in a higher order TFIID core complex containing seven TAFs. TAF8 forms a heterodimer with TAF10 through its HF and proline rich domains, and also interacts with SPT7L through its C-terminal region, and the three proteins form a complex *in vitro* and *in vivo*. Thus, the TAF8-TAF10 and TAF10-SPT7L HF pairs, and also the SMAT complex, seem to be important regulators of the composition of different TFIID and/or STAGA/TFTC complexes in the nucleus and consequently may play a role in gene regulation.

## Introduction

Transcription initiation of protein-coding genes by RNA polymerase II (Pol II) requires the ordered assembly of general transcription factors (GTFs) at the minimal promoter of these genes to form a functional preinitiation complex (PIC). Transcription factor TFIID, comprised of the TATA binding protein (TBP) and series of TBP-associated factors (TAFs) [1], [2], [3], is the GTF that by recognizing the promoter sequences allows the site specific assembly of the PIC. We have previously shown that in HeLa cells different human TFIID complexes, containing or lacking TAF10, exist, which exhibit functionally distinct properties [4], [5]. TAFs are not only integral components of TFIID, but are also found in the yeast Spt-Ada-Gcn5 acetylase (SAGA) coactivator complex [6]. Likewise in mammalian cells, TAFs are shared between TFIID and three very closely related multiprotein complexes, which we refer to as STAGA/TFTC-like complexes: the TBP-free TAF-containing complex (TFTC), the p300/CBP associated factor (PCAF) complex, the GCN5 complex and the SPT3-TAF9-GCN5 containing complex (STAGA) [7], [8], [9]. All of these complexes contain homologues of the yeast histone acetyl transferase (HAT) GCN5, as well as a subset of SPT and ADA proteins, the 400 kDa protein TRRAP, and a number of TAFs (shared TAFs) also found in TFIID. TFTC is structurally similar to TFIID [10], [11], and although devoid of TBP, it is capable of functionally replacing TFIID at both TATA-containing and TATA-less promoters *in vitro* [7].

Electron microscopy, yeast genetics, X-ray crystallography and biochemical experiments have shown that histone fold (HF) motifs mediate many of the subunit interactions within the yeast TFIID and SAGA complexes [12]. In addition, HF-containing TAFs play also roles in promoter recognition and exert coactivator function by interacting and possibly recruiting components of the preinitiation complex [12], [13], [14], [15]. At present five HF-containing TAF pairs have been described or suggested to exist in TFIID: TAF6-TAF9, TAF4-TAF12, TAF11-13, TAF8-TAF10 and TAF3-TAF10 [13], [16], [17], [18], [19]. The mapping of histone-like TAFs in yeast TFIID revealed that these TAF pairs are organized in three distinct lobes within TFIID [20] and not in a single octamer-like structure as previously suggested. Similarly, in SAGA the existence of three putative HF domain-containing heterodimer pairs has been suggested: TAF6-TAF9, TAF10-Spt7p and TAF12-Ada1 [12] and refs. therein). The spatial distribution of TAF5, TAF6 and TAF10 was found similar in SAGA and TFIID and was used as the basis of alignment of the two complexes [20], [21]. The alignment suggests that the 4 nm wide groove of TFIID, which could be involved in DNA binding, is similar to the cleft formed by domains II, III, and IV of SAGA. The location of TAF5 together with the HF domain-containing proteins in both complexes suggests that similarly to TFIID, the WD-40 repeat-containing TAF5 protein that forms the structural backbone of the complex connects the HF-heterodimer pairs within SAGA complexes. Recently, we have shown that the human TAF10 can form three HF pairs with TAF3, TAF8, or SPT7L, which is the human orthologue of yeast Spt7p, also called STAF65γ [15] [22].

*Drosophila* Prodos (PDS) is a protein essential for cell viability that comprises a HF, which selectively heterodimerises with dmTAF10b, but not with dmTAF10 [23]. Consequently it was proposed that PDS is a *Drosophila* TFIID component [23] and has been named dmTAF8 [3]. Recently the human homologue of TAF8 (TAF_II_43) was also described as an integral component of TFIID [13]. Both PDS and human TAF8 are orthologues of mouse Taube Nuss (TBN), which is essential for early embryonic developmental events [24]. Interestingly, *TBN*-/- (*TAF8*-/-) and *TAF10*-/- mice have the same phenotype showing that the lack of either mmTAF8 or mmTAF10 leads to dramatic, but selective, cell death in the inner cell mass [24], [25]. These knock out results further underline the possibility that these two mammalian proteins interact *in vivo* and have similar or related roles in the respective TAF-containing complexes.

Recently we have shown that exogenously expressed TAF10 remains mainly cytoplasmic and leptomycin B does not affect this localisation [15]. By using fluorescent fusion proteins, we showed that TAF10 needs one of its three HF-containing interaction partners (TAF3, TAF8 or SPT7L) to be transported into the nucleus. When the nuclear localisation signals of either TAF8 or SPT7L are mutated, TAF10 remains cytoplasmic, but a heterologous NLS can drive TAF10 into the nucleus. Moreover, TAF10 binding to importin β *in vitro* was dependent on the co-expression of either TAF8 or TAF3, but not SPT7L [15]. These data suggest that a complex network of regulated cytoplasmic associations may exist among these factors, which is important for the assembly of different TFIID and TFTC-type complexes in the nucleus.

Much attention has been focused on the exact subunit composition of multiprotein coregulator complexes with relatively little attention paid to how these complexes are assembled and disassembled in the cell, a theme that appears to involve more dynamism and versatility than previously imagined. In order to further investigate how TAF-interactions regulate the formation of TFIID and TFTC/STAGA, we performed additional proteomic and biochemical analyses to identify in which complexes TAF8 and SPT7L are found. Here we demonstrate that human TAF8 can interact *in vitro* and *in vivo* with TAF10 through its HF and with SPT7L through its C-terminal region. Moreover, we show that TAF8 is absolutely required for the integration of TAF10 in a higher order TAF complex containing seven TAFs. Interestingly, we discovered that TAF8 is not a stable component of TFTC/STAGA complexes, but is present in a novel small TAF complex (SMAT), containing TAF8, TAF10 and SPT7L. The fact that TAF8, TAF10 and SPT7L can never be found together either in TFIID or in TFTC/STAGA-type complexes suggests that SMAT has a separate role in the regulation of transcription. As the expression of TAF8 may regulate the nuclear localization of other TAFs and/or TFTC subunits, as well as specific cell differentiation processes, the SMAT complex may be an important regulator of the composition of different TFIID or TFTC-type complexes in the nucleus and/or cell differentiation processes.

## Results

### Identification of human TAF8 and SPT7L in TFTC preparations

In order to identify new TFTC subunits [7], two protein species migrating around 45 and 65 kDa were excised from a 10% SDS-PAGE and analysed by either microsequencing (45 kDa band) or by MALDI mass spectrometry (65 kDa band). The analysis of the protein species migrating around 45 kDa by Edmann degradation and microsequencing resulted in the following tryptic peptide sequences: (R)EPVSDYQVLR, (K)TGETQSL, (K)DDVS-TFPLIAAR, (K)ENTSVLQQNPSL. Database searches indicated that these peptides all originated from human TAF8. The MALDI mass spectrometry analysis of the 65 kDa protein species identified 5 peptides: (R)YWGEIPISSSQTNR, (R)SSFDLLPR, (R)N-LITAQAQNQQQTEGVK, (K)NPNAPFQIR, (R)HSDPESDFYR (Figure S1). They all corresponded to a 414 amino acid human protein (O94864 and AAG47636; coverage: 14%; average mass accuracy: 30 ppm) that has an overall sequence similarity of 46% to the C-terminal half of yeast Spt7p (Figure S1), a component of the yeast SAGA complex. The identified human protein contains a putative HF motif similar to that of ySpt7p. Thus, following the yeast SAGA subunit nomenclature, we called this human protein Suppressor of Ty 7-like (hereafter SPT7L) [15]. Note however, that human SPT7L lacks the bromodomain present in yeast Spt7p (Figure S1). SPT7L has already been identified as a tumour-rejection antigen in lung adenocarcinoma (named ART1/P17 [26]) and, in good agreement with our results, described also as a subunit of the human STAGA complex (called STAF65γ [22]).

**Figure 1:**
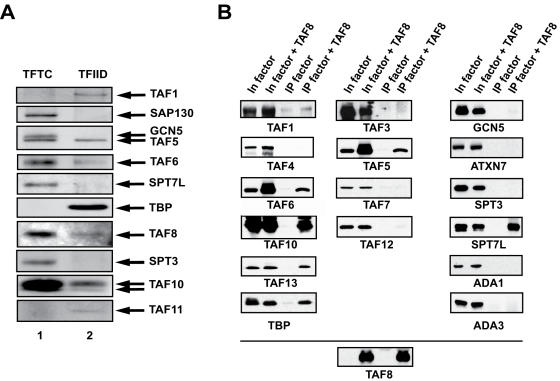
TAF8 is present in both TFIID and TFTC preparations while SPT7L is associated with TFTC. (A) TFIID and TFTC complexes were purified [7], separated by SDS-PAGE and the presence of different subunits were analysed by Western blot using the indicated antibodies. (B) TAFs, TBP (left panels) and certain TFTC subunits (right panels) were individually expressed (In factor) or coexpressed with TAF8 (In factor+TAF8) in Sf9 cells as indicated and WCEs were made. TAF8-containing complexes were immunopurified (IPed) with an anti-TAF8 mAb (2TAU 2B8). Protein expression in the input fractions and TAF8-bound proteins were analysed by western blot with the indicated antibodies. In each experiment, the expression and the IP efficiency of TAF8 was the same as shown in a single representative experiment (lower panel).

To verify the presence of TAF8 and SPT7L in TFTC preparations, we raised anti-peptide rabbit polyclonal and mouse monoclonal antibodies (mAbs) against TAF8 and hSPT7L (see Materials and Methods). These antibodies recognized both recombinant and endogenous TAF8 and SPT7L proteins, respectively and were then used to verify the presence of TAF8 and SPT7L in purified human TFTC and TFIID preparations by Western blot analysis. TAF8 was present in TFTC, and also in TFIID (Fig. 1A). In agreement with previous data [22], SPT7L was present in TFTC, but not in highly purified TFIID preparations (Fig. 1A). These results suggest that human TAF8 is a component of both TFIID and TFTC (see also below), while SPT7L is a TFTC specific subunit.

### *In vitro* TAF8 interacts with several TAFs, but only with SPT7L among the TFTC/STAGA specific subunits

First, we wanted to determine with which TFIID or TFTC subunits TAF8 would interact. To this end, TAF8 was co-expressed with either one of the human TAFs or TBP (two left columns in Fig. 1B), or specific human TFTC/STAGA subunits (right column in Fig. 1B) using a baculovirus expression system in Sf9 insect cells [27], [28]. Each factor was also expressed alone as a control. From these cells total protein extracts were made, proteins were immunoprecipated (IPed) with an anti-TAF8 mAb (2TAU 2B8), and bead-bound proteins were analysed by Western blot. This experiment showed that *in vitro* TAF8 could interact with several HF-containing TAFs (TAF6, TAF10 and TAF13), with the WD40 containing TAF5, and TBP (Fig. 1B, two left columns). Among the tested TFTC specific subunits, only SPT7L interacted stably with TAF8 (Fig. 1B, right column and see Fig. 1C). These proteins were not IPed from Sf9 extracts where they were expressed individually without TAF8 (Fig. 1B).

**Figure 2:**
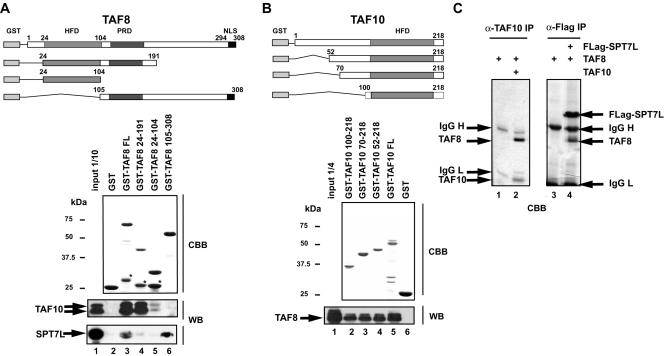
TAF8 interacts with TAF10 through its HF domain, while it interacts with SPT7L through its C-terminal region. GST-TAF8 (A) and GST-TAF10 (B) full length (FL) or the corresponding deletion mutants were immobilised on glutathion-sepharose beads (as indicated), washed and the bound GST-fusion proteins were separated by SDS-PAGE and visualized by Coomassie brilliant blue staining (CBB). (*) labels premature termination products. Equal amounts of the indicated baculovirus infected Sf9 whole cell extracts (WCEs) were incubated with each resin, washed and the bound proteins analysed by western blot (WB). The positions of molecular weight markers are indicated in kDa. HFD: histone fold domain; PRD: proline rich domain; NLS: nuclear localization signal. (C) TAF8 was expressed alone (lane 1 and 3), coexpressed with TAF10 (lane 2) or with Flag-SPT7L (lane 4) in Sf9 cells using the baculovirus system. From these cells WCEs were prepared and proteins immunoprecipitated with the indicated antibodies. Bound proteins were separated by SDS-PAGE and visualized by Coomassie brilliant blue staining (CBB). The co-immunoprecipitation between TAF8 and TAF10 as well as TAF8 and SPT7L indicates strong stoichiometric interactions between these proteins.

Since the interaction between TAF8 and TAF10 has been confirmed in several different species [13], [15], [18], [23] we wanted to fine map the minimal regions of either TAF8 or TAF10 that are sufficient for this interaction. To this end TAF8 and its truncated mutants were expressed in *E. coli* as GST fusion proteins, bound to glutathione sepharose beads, and used for pull-down experiments with baculovirus overexpressed TAF10 (Fig. 2A). TAF10 is weakly binding to the formerly defined HF domain of TAF8 (GST-TAF8 24-104; Fig. 2A lane 5), however this binding is significantly enhanced when the proline rich domain of TAF8 is present in the mutants (compare lane 4 with 5). Note that the C-terminal half of TAF8 lacking the HF, but containing the proline rich domain does not bind TAF10 (lane 6). When mapping the minimal interaction domain of TAF10 with TAF8 in a similar GST pull-down experiment we found that amino acids 100 to 218 of TAF10, containing its evolutionary conserved region including the putative HF, are sufficient to interact with TAF8 (Fig. 2B). These results demonstrate that TAF8 and TAF10 interact through their HF domains, and this interaction is strengthened by additional contacts or potential conformational changes mediated by the proline rich domain of TAF8.

Surprisingly, baculovirus expressed TAF8 interacts very strongly with SPT7L (Fig. 2C). Since this is the only TFTC specific subunit that interacts with TAF8 among the tested subunits, and because SPT7L contains also a putative HF, we mapped the domain of TAF8 that is involved in the interaction with SPT7L. To this end the same TAF8 mutants were used that were described above (Fig. 2A lowest WB panel). Interestingly, the TAF8 HF and proline rich domains do not seem to be involved in the interaction between the GST-TAF8 mutants and the baculovirus expressed SPT7L (lanes 4 and 5). Contrary to our original expectation, the region of TAF8 that interacts with SPT7L is at the C-terminal end between amino acids 194 and 308 (Fig. 2A lane 6). These results suggest that TAF10 and SPT7L do not bind to the same region of TAF8.

### TAF8 and SPT7L interact in the cell

**Figure 3:**
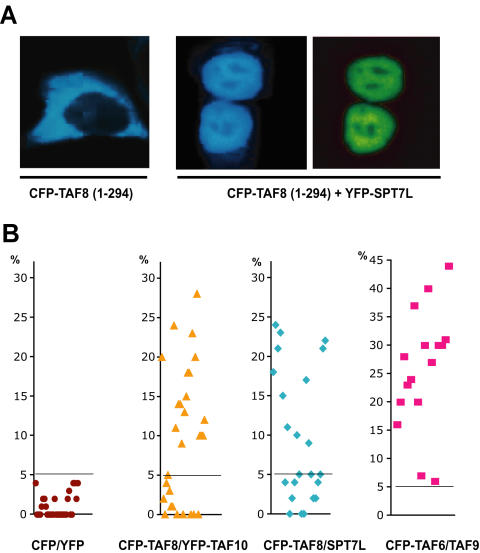
TAF8 and SPT7L interact in vivo (A) The nuclear localization of TAF8 lacking the NLS [TAF8(1-294] depends on its *in vivo* interaction with SPT7L. HeLa cells were co-transfected with the indicated CFP- and YFP-containing expression vectors and localization of the expressed proteins were visualised by fluorescence microscopy. The images shown in each panel are representative of all the transfected cells. (B) Sensitized emission of YFP fusion proteins due to FRET was measured in two different experiments in the nucleus of 25 individual HeLa cells transfected with the indicated combinations of vectors expressing YFP and CFP fusion proteins. The mean value of FRET efficiency (in%) over the entire nucleus in each cell was calculated as described in the Materials and Methods. A threshold was set to 5%, above the highest value of the negative control CFP/YFP (see horizontal line in each graph), and for the other pairs only values above this level were averaged. The average value of the negative control is 1.03%. The average value for each pair is the following: CFP-TAF8/YFP-TAF10 = 16.9%; CFP-TAF8/YFP-SPT7L = 15.8%; CFP-TAF6/YFP-TAF9 = 27.1%. Note that the scale for the TAF6/TAF9 is different.

To verify the surprising novel interaction found between TAF8 and SPT7L *in vivo*, we expressed a truncated form of TAF8 that lacks its C terminal nuclear localization signal (NLS). We then asked the question whether the interaction between SPT7L and TAF8 would be maintained and thus SPT7L would take the TAF8 mutant into the nucleus (Fig. 3A). The cells were first transfected with a construct expressing a truncated version of TAF8(1-294) lacking the NLS of TAF8 as a CFP fusion protein individually (Fig. 3A). When the cellular localisation of this fusion protein was analysed, as expected, the TAF8(1-294) mutant localised exclusively to the cytoplasm (Fig. 3A). Next, we co-expressed the CFP-TAF8(1-294) mutant with YFP-SPT7L in HeLa cells and tested their localisation. In the presence of SPT7L the TAF8(1-294) mutant became nuclear (Fig. 3A), indicating that the two proteins interact in the cell.

Next, we aimed to further analyse the TAF8/TAF10 and TAF8/SPT7L interactions in living cells and compare them to that of a previously characterised TAF pair. To this end, we measured FRET by the sensitized emission method in living cells. HeLa cells were transfected with constructs expressing the YFP/CFP, the CFP-TAF8/YFP-TAF10 and the CFP-TAF8/YFP-SPT7L pairs, as well as the TAF6/TA9 HF pair as a positive control. Mean FRET efficiencies were measured in the nucleus of 25–30 individual cells for each combination. When measuring FRET with the control CFP/YFP pair, where the two fluorescent proteins are supposed to interact only randomly, we obtained FRET efficiencies varying between 0 and 4% in all cells, with an average of 1.03% (Fig. 3B). Thus, in all the other transfections we have only considered cells with values above the 5% threshold as positives. Using these criteria, both the TAF8/TAF10 and TAF8/SPT7L interactions resulted in similar FRET efficiencies, at around 16–17% (Fig. 3B). The positive control TAF6/TAF9 HF pair gave 27.1% FRET efficiency (Fig. 3B). The above data indicate that TAF8-TAF10 and TAF8-SPT7L pairs form *in vivo*.

### TAF8 is absolutely required for the incorporation of TAF10 into a higher order TAF complex

**Figure 4:**
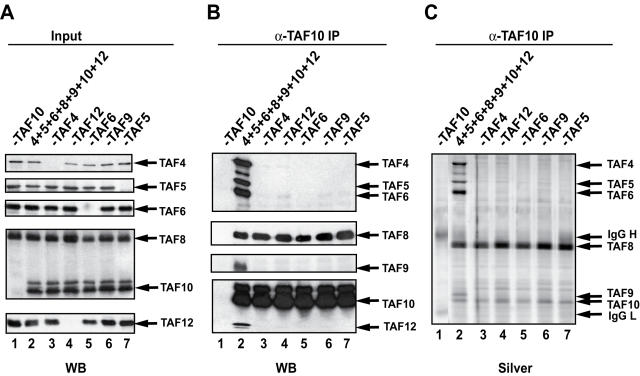
The TAF8-TAF10 HF pair incorporates into a higher order TFIID subcomplex only if another five TAFs are simultaneously present. (A) Either seven TAFs (TAF4, -5, -6, -8, -9, -10 and -12) or only six of them (each time only the omitted TAF is indicated compared to the seven-TAF complex) were coexpressed in Sf9 cells and WCEs made. Protein expression in the WCEs was verified by western blot analysis (WB). Note that in the seven-TAF complex each TAF is indicated with only its corresponding number according to the TAF nomenclature [3]. (B–C) TAF10-containing complexes were purified from the WCEs with an anti-TAF10 mAb (1H8). Bound complexes were eluted by peptide competition and analysed either by western blot (WB) with the indicated antibodies (B) or by silver nitrate staining (Silver) (C).

Over the past years our efforts to build complete or recombinant human TFIID complexes containing TAF10, using the baculovirus overexpression system, have been without any success. When TAF10 was overexpressed with all the known TFIID subunits, except for TAF8, TAF10 was unable to interact with any of the other TAFs or TBP (Table 1 line 1; and data not shown). However, when TAF8 was also included in the coinfection where in total 13 subunits of the TFIID complex were expressed; we were able to coprecipitate TAF10 with six other TAFs (TAF4, -5, -6, -8, -9, -10 and –12) containing three HF pairs (TAF4–12, TAF6–9, and TAF8–10) and the WD40 repeat-containing TAF5 (Table 1 see second line). When only these seven TAFs were coexpressed they, indeed, formed a single complex (Figure 4B and C lanes 2; and Table 1 fourth line), as demonstrated also by size fractionation of the immunopurified material [28]. Interestingly however, when any of the seven TAFs was left out from the co-infection, this recombinant seven TAF-containing complex did not form and TAF10 interacted only with TAF8 (Fig. 4 and Table 1 compare lines 3–4). In good agreement with the high specificity interactions occurring in this seven TAF complex, the TAF8-TAF10 HF pair did not interact with any other HF-containing TAF pair individually or in combination (Table 1, lines 5–8). Moreover, when these individual TAF pairs were coexpressed with the WD40-containing TAF5, no interactions with the TAF8–TAF10 pair were detected (Table 1, lines 9–13). Similarly, no interactions between TAF8–TAF10 HF pair and the other factors could be detected when TBP, TAF1, TAF2 and TAF7 were co-expressed with the above tested TAF combinations (see Table 1, lines 14–24). These experiments together demonstrate that TAF8 is necessary for the incorporation of TAF10 in a higher-order seven TAF complex and that the simultaneous incorporation of all of these factors (TAF4, -5, -6, -8, -9, and -10) is absolutely required to form this stable recombinant TFIID subcomplex.

**Table 1:** 

	Extracts														Anti-TAF10 IPs													
	TAF10	TAF8	TBP	TAF1	TAF2	TAF4	TAF5	TAF6	TAF7	TAF9	TAF11	TAF12	TAF13	SPT7L	TAF10	TAF8	TBP	TAF1	TAF2	TAF4	TAF5	TAF6	TAF7	TAF9	TAF11	TAF12	TAF13	SPT7L
**1**	**+**		**+**	**+**	**+**	**+**	**+**	**+**	**+**	**+**	**+**	**+**	**+**		**+**		**-**	**-**	**-**	**-**	**-**	**-**	**-**	**-**	**-**	**-**	**-**	
**2**	**+**	**+**	**+**	**+**	**+**	**+**	**+**	**+**	**+**	**+**	**+**	**+**	**+**		**+**	**+**	**-**	**-**	**-**	**+**	**+**	**+**	**-**	**+**	**-**	**+**	**-**	
**3**	**+**					**+**	**+**	**+**		**+**		**+**			**+**					**-**	**-**	**-**		**-**		**-**		
**4**	**+**	**+**				**+**	**+**	**+**		**+**		**+**			**+**	**+**				**+**	**+**	**+**		**+**		**+**		
**5**	**+**	**+**						**+**		**+**					**+**	**+**						**-**		**-**				
**6**	**+**	**+**									**+**		**+**		**+**	**+**									**-**		**-**	
**7**	**+**	**+**				**+**						**+**			**+**	**+**				**-**						**-**		
**8**	**+**	**+**						**+**		**+**	**+**		**+**		**+**	**+**						**-**		**-**	**-**		**-**	
**9**	**+**	**+**					**+**								**+**	**+**					**-**							
**10**	**+**	**+**				**+**	**+**					**+**			**+**	**+**				**-**	**-**					**-**		
**11**	**+**	**+**					**+**	**+**		**+**					**+**	**+**					**-**	**-**		**-**				
**12**	**+**	**+**					**+**				**+**		**+**		**+**	**+**					**-**				**-**		**-**	
**13**	**+**	**+**					**+**	**+**		**+**	**+**		**+**		**+**	**+**					**-**	**-**		**-**	**-**		**-**	
**14**	**+**	**+**							**+**						**+**	**+**							**-**					
**15**	**+**	**+**				**+**		**+**	**+**	**+**	**+**	**+**	**+**		**+**	**+**				**-**		**-**	**-**	**-**	**-**	**-**	**-**	
**16**	**+**	**+**			**+**										**+**	**+**			**-**									
**17**	**+**	**+**			**+**			**+**		**+**	**+**		**+**		**+**	**+**			**-**			**-**		**-**	**-**		**-**	
**18**	**+**	**+**			**+**		**+**	**+**	**+**	**+**	**+**		**+**		**+**	**+**			**-**		**-**	**-**	**-**	**-**	**-**		**-**	
**19**	**+**	**+**	**+**	**+**											**+**	**+**	**-**	**-**										
**20**	**+**	**+**	**+**	**+**					**+**						**+**	**+**	**-**	**-**					**-**					
**21**	**+**	**+**	**+**	**+**			**+**		**+**						**+**	**+**	**-**	**-**			**-**		**-**					
**22**	**+**	**+**	**+**	**+**	**+**		**+**		**+**						**+**	**+**	**-**	**-**	**-**		**-**		**-**					
**23**	**+**	**+**	**+**	**+**	**+**		**+**	**+**	**+**	**+**					**+**	**+**	**-**	**-**	**-**		**-**	**-**	**-**	**-**				
**24**	**+**	**+**	**+**	**+**	**+**		**+**	**+**	**+**	**+**	**+**		**+**		**+**	**+**	**-**	**-**	**-**		**-**	**-**	**-**	**-**	**-**		**-**	
**25**	**+**	**+**				**+**	**+**	**+**		**+**		**+**		**+**	**+**	**+**				**+**	**+**	**+**		**+**		**+**		**-**
**26**	**+**					**+**	**+**	**+**		**+**		**+**		**+**	**+**					**-**	**-**	**-**		**-**		**-**		**+**
**27**	**+**	**+**												**+**	**+**	**+**												**+**

On the left half of the Table are shown the co-infections with various TAF, TBP or SPT7L expressing baculoviruses in Sf9 cells. (**+)** indicates that the given factor was expressed in the prepared whole cell extracts (WCEs). On the right half of the Table are shown those factors (+), which were co-IP-ed from these extracts by an anti-TAF10 IP using the 1H8 mAb. Factors, which were present in the input extract but did not co-purify with TAF10 are marked with (-).

Moreover, the comparison of the pair wise interaction profile obtained between TAF8 and many of its potential partners (Fig. 1B) with that obtained between the TAF8-TAF10 HF pair and a large combination of TAFs (shown in Fig. 4 and Table 1), indicates that interactions within the TFIID complex amongst TAFs or TAF pairs become more and more specific when more interaction partners are coexpressed together. These simultaneous, highly specific multiple interactions seem to eliminate weaker, promiscuous interactions [29] amongst TAFs or HF-containing TAF pairs.

### TAF8 is not a subunit of TFTC/STAGA type complexes, but is present in TFIID and a novel TAF8-, TAF10- and SPT7L-containing complex

**Figure 5:**
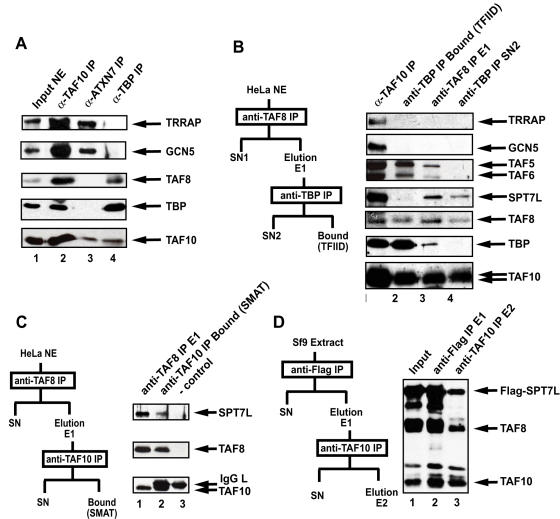
TAF8 is not a subunit of TFTC/STAGA type complexes, but is present in TFIID and a novel TAF8-, TAF10- and SPT7L-containing complex that can also be formed *in vitro*. (A) The indicated transcription factors were immunoprecipitated using specific mAbs (1H8 anti-TAF10; 2A10 anti-ATXN7 and 2C1 anti-TBP) from HeLa cell nuclear extract (NE) and eluted by an excess of peptides against which the mAbs were raised. Eluted protein complexes were separated by SDS-PAGE and analysed by western blot with the indicated antibodies. (B) TAF8-containing protein complexes were purified from HeLa NE according to the scheme shown on the left of the panel. The eluate obtained after the first anti-TAF8 IP using the 2TAU 2B8 mAb (anti-TAF8 IP E1; lane 3) and the supernatant obtained after the TAF8-containing complexes were depleted in TFIID using the anti-TBP 2C1 mAb (SN2; lane 4). They were separated along with complexes obtained after an anti-TAF10 IP (TFIID and TFTC together; lane 1) or with a highly purified TFIID fraction (lane 2) on SDS-PAGE and analysed with the indicated antibodies by western blot. (C) TAF8 containing complexes were purified from HeLa NE according to the scheme on the left of the panel. TAF10-containing complexes were re-precipitated from the eluate obtained after the first anti-TAF8 IP (E1, lane 1) extensively washed and loaded after boiling the beads (lane 2) with loading buffer on a SDS-PAGE. The migration of the antibody alone (-control) is shown in lane 3. Proteins were analyzed with the indicated antibodies by western blot. (D) TAF8, TAF10 and Flag-SPT7L were co-expressed in Sf9 cells. WCEs were made and proteins were subjected to two successive immunoprecipitations and elutions by peptide competition according to the schemes shown on the left of the panel. Input and eluted protein complexes were separated by SDS-PAGE and analysed with the indicated antibodies by western blot.

To further characterize the association of TAF8 with TFTC/STAGA complexes we first immunoprecipitated TFTC/STAGA complexes with an antibody raised against ATXN7, a recently characterized subunit of TFTC/STAGA [30], [31], and looked for the presence TAF8 in these complexes by western blot analysis (Fig. 5A lane 3). Surprisingly, and in contradiction with the initial mass spectrometry data, no TAF8 could be detected in these ATXN7-containing TFTC/STAGA complexes. However, we detected TRRAP, GCN5, TAF10 by western blot (lane 3) and also ATXN7, TAF5L, TAF6L, ADA1 (STAF42) and SPT7L by mass spectrometry (data not shown). To further verify this surprising observation we carried out a reciprocal anti-TAF8 IP using the 2TAU 2B8 mAb and tested the presence of TFTC/STAGA or TFIID subunits in this immunopurified fraction that contains only TAF8-associated proteins. By western blot analysis we detected in this fraction TFIID subunits such as TAF5, TAF6, TAF8, TAF10 and TBP, but no TRRAP and GCN5, which are specific TFTC/STAGA components (Fig. 5B lane 3). This finding seemed to be in agreement with the results of the above ATXN7 IP and with those of Guermah et al. [13] showing that TAF8 is not a STAGA component. Unexpectedly however, we detected SPT7L, a TFTC/STAGA component, in the TAF8 IP. One way to explain the presence of SPT7L alone in the TAF8 IP, without other TFTC components, is that TAF8 *in vivo* forms also a complex with SPT7L (as we have shown above by different interaction experiments) and that this complex is not present in TFTC. To verify this hypothesis we depleted TBP-containing (TFIID) complexes from the immunoprurified TAF8-containing complexes by using an antibody raised against TBP. The depletion of TFIID from this fraction (anti-TBP IP SN2 in the scheme of Fig. 5B) was complete, since we could not detect TAF5, TAF6 and TBP in this SN2 fraction, however we could still detect SPT7L, TAF8 and TAF10 (Fig. 5B lane 4). To confirm that the three proteins are present in the same complex, we performed a two-step immunoprecipitation from HeLa NE. We first purified the TAF8-containing complexes and then reprecipitated TAF10 from the eluate of the first IP with an anti TAF10 mAb (Fig. 5C). TAF8, TAF10, SPT7L could be co-precipitated in the second IP, showing that they are present together in a complex. Mass spectrometry analysis of the SN2 fraction in Fig. 5B did not reveal the presence of additional known TFIID or TFTC subunits. These findings suggest that in the cells a protein complex exists containing SPT7L, TAF8 and TAF10, but no other TFIID or TFTC specific subunits. The existence of such a SPT7L-, TAF8- and TAF10-containing complex that is different from all the previously described TAF-containing complexes, also explains why we have originally identified TAF8 in our TFTC preparation (see Discussion).

### The TAF8-, TAF10- and SPT7L-containing complex can also be formed *in vitro*

Since TAF8, TAF10 and SPT7L could be found in a complex together in endogenous HeLa nuclear extracts and since TAF8 interacts with both TAF10 and SPT7L, [15] we verified whether these three proteins can form a trimeric complex *in vitro*. Flag-SPT7L TAF8- and TAF10- were coexpressed in Sf9 cells and purified by a double IP, either with an anti-FLAG IP followed by an anti-TAF10 IP (Fig. 5D) or *vice versa*, with an anti-TAF10 IP followed by an anti-FLAG IP (data not shown). TAF8-, TAF10- and SPT7L could be co-immunoprecipitated in both cases (Fig. 5D lane 3, and data not shown), confirming that these three proteins can form a recombinant complex.

Since all the three subunits of this small TAF8-, TAF10- and SPT7L-containing complex (SMAT) are never present at the same time either in TFIID or in STAGA, we hypothetised that SMAT could be a storage complex for these factors, which upon getting incorporated into the appropriate complex would inflict on whether a TFIID or STAGA/TFTC is to be assembled. To test this hypothesis, we coexpressed all the described subunits of the seven TAF complex with SPT7L or we replaced TAF8 in the seven TAF complex with SPT7L. When SPT7L was coexpressed with the subunits of the seven TAF complex, we could still immunopurify with an anti-TAF10 mAb the seven TAF complex, but SPT7L did not incorporate in this complex (Table 1 line 25). Moreover, when TAF8 was left out from the seven TAF complex and replaced by SPT7L, TAF10 interacted only with SPT7L, but the other TFIID TAFs did not form a complex with the TAF10-SPT7L heterodimer (Table 1 line 26). Together these data indicate that when all the subunits of a potential TFIID core complex are present, SPT7L cannot turn the formation of the TFIID core into a STAGA/TFTC core. Conversely, when the formation of a TFTC core is initiated by the formation of a TAF10-SPT7L HF pair, the TFIID core cannot assemble (see also below and Discussion).

### The novel TAF8-, TAF10- and SPT7L-containing complex has a size of 300–400 kDa

**Figure 6:**
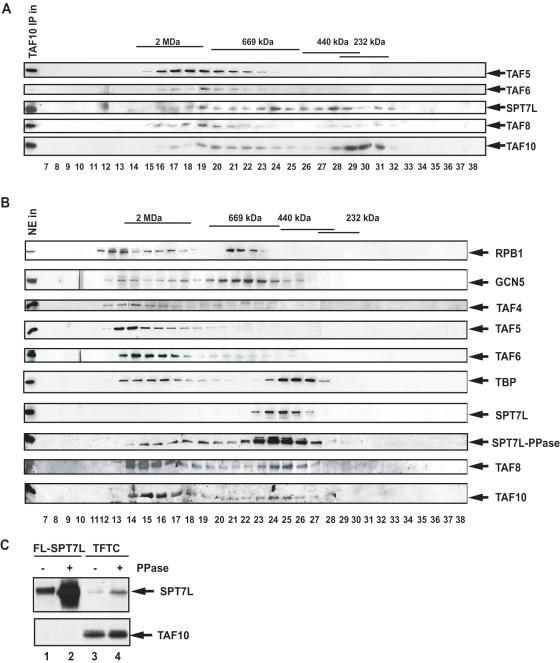
The TAF8-, TAF10- and SPT7L-containing complex has a size of about 300–350 kDa and may exist *in vivo* in crude nuclear extracts. Immunupurified and eluted TAF10-containing complexes (TAF10 IP) (A), or HeLa nuclear extract (NE) (B) (50 µl) were injected on a Superose 6 size exclusion chromatography column using the SMART FPLC system (Pharmacia) and separated. Portions of the input (in; 5 µl) and every fraction from 7 to 38 (20 µl) were analysed by western blot (as indicated). Fraction numbers are shown under each lane and the elution profile of known molecular mass markers is indicated above the panels. To detect the non-phosphorylated form of SPT7L, 20 µl from each fraction was first phosphatase treated for 30 min at 37°C, then separated by SDS-PAGE and analyzed by western blot with the anti-SPT7L mAb (panel SPT7L-PPase). (C) Baculovirus expressed SPT7L in WCE (lane 2) and purified TFTC (lane 4) were treated with phosphatase (PPase) for 30 min at 37°C, separated on SDS-PAGE and analysed by western blot with the anti-SPT7L mAb (15) (upper panel), and an anti-TAF10 mAb (lower panel). The corresponding mock treated fractions are shown in lanes 1 and 3, respectively.

To determine the size of the SMAT complex, we first immunopurified all the TAF10-containing complexes from HeLa NE by an anti-TAF10 IP and the eluted material was immediately injected on a Superose 6 size exclusion chromatography column. Testing the elution profile of TAF8-, TAF10- and SPT7L we observed that these proteins eluted mainly in two peaks: one between 1 and 2 MDa and a second around 300–400 kDa (Fig. 6A). The broad elution profile of TAF10 suggests that this protein as well as proteins associated with it is present in several different complexes in human HeLa cells.

To further verify the presence of these different smaller TAF-containing complexes in HeLa cell extracts, we fractionated a crude HeLa cell nuclear extract (NE) on the same Superose 6 gel filtration column (Fig. 6B). The two peaks obtained for the largest subunit of RNA Pol II (RPB1) correspond to the previously described holo-polymerase (about 2 MDa) and core polymerase complexes (650 kDa) [32], as well as the two peaks of TBP correspond to the previously described TBP-containing complexes [33]. The correct separation of these complexes confirmed that our NE contained intact complexes. Interestingly, while TAF4, TAF5 and TAF6 eluted from the column as a single peak in the 1–2 MDa range, TAF8 and TAF10 were detected in at least two peaks eluting from the column in a molecular range of 1–2 MDa and about 400–500 kDa. Surprisingly, SPT7L was detected in only a peak around 450–500 kDa. Our anti-SPT7L mAb recognizes mainly the phoshatase treated form of recombinant SPT7L in western blot analysis (Fig. 6C lanes 1 and 2) suggesting that one or several of the amino acids in the SPT7L epitope (QSPDDSDSSYGSHSTDSLM) are phosphorylated. Endogenous SPT7L is also phosphorylated in the TFTC complex because SPT7L can be better detected following phosphatase treatment of the complex by western blot analysis (Fig. 6C, compare lane 3 and 4). To verify whether the lack of SPT7L detection in the Superose 6 fractions containing the higher molecular weight complexes was due to the phophorylation of SPT7L, each fraction was phosphatase treated and then analysed again by western blot. When comparing the two series of blots (SPT7L and SPT7L-PPase panels in Fig. 6B) it became clear that SPT7L is present also in higher molecular weight complexes as expected, but that the majority of SPT7L peaks around 450–500 kDa. These data together suggest that the NE fractions around 400–500 kDa contain the above-described TAF8-, TAF10- and SPT7L-containing complex (Fig. 6B fractions 24–27). Interestingly, the elution profile of each tested factor is shifted towards higher molecular weights in the fractionation of crude NE when compared to the separation of the IP-ed complexes (compare panel A and B in Fig. 6). This may be explained by the presence of additional loosely associated proteins in the complexes (i.e. chaperones and or transcription activators) found in the crude NE, which proteins, due to the stringent washes during the immunopurification (500 mM KCl and 0.1% NP40) could dissociate. These data together demonstrate the existence of a TAF8-, TAF10- and SPT7L-containing SMAT complex in HeLa cells.

## Discussion

### TAF8 is not a STAGA/TFTC component

The main finding of this study is the discovery of a novel TAF-containing complex, that we call SMAT. SMAT is composed of three HF-containing factors, TAF8, TAF10, SPT7L, and potentially as yet unidentified proteins. Despite that we identified TAF8 from our original TFTC preparations, using TFTC/STAGA specific antibodies we show here that TAF8 was not a component of the large 2 MDa TFTC/STAGA complex, but it was present in a smaller complex that copurified with the TFTC preparation. As the TFTC fraction was the supernatant of a second TBP IP step [7] it remained possible that this TFTC preparation may contain several different TAF10-containing complexes. The gel filtration of these endogenous complexes, as well as the size separation of the crude NE, on a Superose 6 column clearly shows that in HeLa cells more TAF10-containing complexes exist than originally expected. Our results presented here indicate that TFTC is a mixture of STAGA, SMAT and possibly other TAF10-containing complexes. Moreover, it is important to realize that SMAT cannot be a break down product of either TFIID, or STAGA because TFIID does not contain SPT7L and because STAGA does not contain TAF8 (13 and this study).

### What is the role of the novel TAF8-, TAF10-, SPT7L-containing complex?

One possible role of SMAT could be that it controls the nuclear equilibrium between TFIID and TFTC/STAGA-type complexes. In yeast TFIID function seems to predominate approximately 90% of tested genes and SAGA function is important at approximately 10% of the yeast genome. TFIID-dependent genes seem to be more ubiquitously expressed, SAGA-dependent ones on the other hand, seem to be largely stress induced [34]. Thus, it is conceivable that a TAF8-, TAF10-, SPT7L-containing complex by releasing or sequestering TAF8 and/or SPT7L may regulate the quick assembly or disassembly of complete TFIID or TFTC/STAGA-type complexes in response to stress or once the stress is over.

The fact that the TAF8-TAF10 HF-containing heterodimer does not interact with any other TFIID subunit individually or in combination to form a partial or complete TFIID complex (Table 1), unless TAF4, TAF5, TAF6, TAF9 and TAF12 are simultaneously present (Fig. 4), suggests a high degree of regulation in the assembly of the TAF-containing complexes. Wright et al. (2006) recently suggested the existence of a stable *Drosophila* core TFIID subcomplex, consisting of TAF4, TAF5, TAF6, TAF9 and TAF12 [35]. The formation of the stable seven-TAF complex is consistent with the physiological existence of a TFIID core that, according to our results, would also incorporate an additional HF pair, the TAF8-TAF10 pair. As previously suggested [28] the accretion of a core with either TFIID- or STAGA-specific subunits would commit the assembly process into the formation of one complex or the other. We show here that, in addition to SMAT, the TAF8-TAF10 pair is only present in TFIID, while the TAF10-SPT7L pair is exclusively present in STAGA. Moreover, our reconstitution experiments suggest that the formation of one of these dimers is one of those points, where the assembly of TFIID or STAGA may bifurcate. Our observations suggest that the formation of SMAT, in which the TAF8-TAF10 and TAF10-SPT7L HF pairs are connected by a TAF8-SPT7L interaction, would block the incorporation of the TAF8-TAF10 or TAF10-SPT7L hetrodimers in TFIID or in STAGA type complexes, respectively. Thus, the expression levels of these factors in the cell and especially their quantity in the nucleus may thus be crucial for the formation of the respective complexes.

Moreover, it is possible that cellular signals induced by stress or other stimuli induce a cascade of events that would posttranslationally modify one or several components of the TAF8-, TAF10-, SPT7L-containing complex, and this modification would then release factors from this complex. In agreement with such a model, SPT7L is phosphorylated in STAGA/TFTC, but not in SMAT. Such modifications together with our previous observations that TAF10 needs one of its three HF partners for entering into the nucleus [15], may participate in the regulation of the assembly of TFIID or TFTC/STAGA-type complexes and thus play a role in the regulation of gene expression.

Alternatively, this TAF8-, TAF10-, SPT7L-containing complex may also be independently recruited to promoters of specific genes to regulate gene expression in addition to the already known TAF-containing complexes. Such a scenario would possibly also explain why the detectability of a given TAF versus others or TBP varies between 1 and 100 arbitrary units at different active enhancers and promoter regions when tested by chromatin immunoprecipitation [36], [37]. Smaller TAF-containing complexes may be recruited to promoters by activators, and known large TAF-containing complexes (i.e. TFIID and TFTC/STAGA) would only be assembled from such smaller complexes at promoters. Alternatively, it is also conceivable that following activation of transcription and PIC formation, TFIID and/or TFTC/STAGA would be destabilised and they would leave the promoter as smaller subcomplexes and not as a single big unit. In agreement with these hypotheses it has been observed *in vivo* that two different components of the TFTC/STAGA complex (TRRAP and GCN5) arrive one after the other, and not together, to activated promoters [38], and similarly that TRRAP and GCN5 stay longer at promoters than other TFTC/STAGA subunits (i.e. TAF9 or TAF10) following gene activation [39]. All these *in vivo* observations are in favour of a model, in which these large multiprotein complexes are dynamically and probably constantly assembled and dissociated in the cell nucleus depending on many different cellular requirements.

The SMAT complex containing two HF pairs may form a tetrameric, TAF10-TAF8-SPT7L-TAF10, or an octameric structure, (TAF10-TAF8-SPT7L-TAF10)_2_, similar to that formed by the four histones. The *in vitro* and *in vivo* interaction between TAF8 and SPT7L, which does not involve the HF domain of either of these proteins, explains how the TAF8-TAF10 and the SPT7L-TAF10 HF pairs interact in SMAT. If organized in a histone octamer like structure, the (two HF pairs)x2 would have a molecular weight of about 350 kDa that would be close to what we have determined by gel filtration (300–400 kDa).

### The *in vivo* assembly of TAF-containing complexes

Our data also demonstrate that TAF-containing complexes are not randomly formed, since no monomeric TAFs, or small subcomplexes, containing for example TAF4, TAF5 or TAF6 were detected in HeLa NE (Fig. 6B). A cellular surveillance mechanism by which the cell could distinguish between intact and partial multiprotein complexes has not yet been described. Thus, we believe that TAFs have a strong and intrinsic propensity for association with other TFIID TAFs (and/or STAGA subunits in the case of shared TAFs) and, once inside the nucleus (or even before), a TAF would shortly be captured by other specific subunits. This immediate recruitment would automatically gather the TAFs or STAGA subunits into higher molecular weight complexes (probably towards the formation of TFIID and TFTC/STAGA complexes) and thus any TAF-containing complex would, in the end, arise by a sort of self-assembly. This model is in good agreement with the self-assembly of the seven-TAF complex (Fig. 4 and Table 1) in which each subunit is needed to form the complex. However, posttranslational modifications, which occur *in vivo,* but are missing or different in our *in vitro* system, may be responsible for lack of assembly of the complete TFIID (Table 1, line 2). Alternatively, other mechanisms, such as energy dependent chaperone activities may be required for the completion of a seven-TAF-like subcomplex into TFIID.

Here we describe a novel TAF-containing complex and hypothesize how HF-containing TAF pairs can regulate the topology and possibly the local assembly of the different TAF-containing complexes in which they are incorporated. Thus, this study suggests that possibly more TAF-containing complexes exist in the cells than originally described. Whether such complexes exist for most of the known histone-fold containing TAF pairs during cellular differentiation or metazoan development remains to be answered.

## Materials and Methods

### Plasmid constructions and cell transfections

The eukaryotic expression plasmids for TAF8, TAF10, and SPT7L, the baculovirus expression vectors for TAF3, TAF4, TAF5, TAF6, TAF8, TAF9, TAF10, TAF12, as well as for SPT7L and the YFP and CFP vectors for TAF8, TAF10 and SPT7L have been previously described in [4], [15], [27], [28], [40]. The full length ATXN7 cDNA fused to a sequence encoding a FLAG epitope was excised from the pc7NFL [30] and cloned in the pVL1393 baculovirus vector. The ADA1 cDNA was amplified from a HeLa cDNA library using complementary oligonucleotides and then cloned into the Nde I and Bgl II sites of the pSK277 baculovirus expression vector. The mouse ADA3 cDNA was excised from the pSG5-ADA3 vector [41] and cloned in the Nde I-Xho I sites of the pSK277 baculovirus vector. The hGCN5 cDNA was excised from the pCDNA3-GCN5 vector [42] and cloned in the Nde I-Xba I sites of the pSK277 baculovirus vector. The human SPT3 cDNA was excised from the pGEX-4T2-SPT3 vector [43] and cloned in the Nde I-Bam HI sites of the pSK277 baculovirus vector. The prokaryotic expression vectors for TAF10 have been described in [4] and for TAF8 they were constructed by PCR amplifying the mouse coding sequence and inserting it into a pGEX-4T3 expression vector between the Eco RI and the Xho I sites. Deletion mutants were similarly generated by PCR and they were inserted into pGEX-2T as Bgl II-Eco RI fragments at the Bam HI and Eco RI cleavage sites. All constructs were verified by sequencing.

1,5×10^5^ HeLa cells were transfected by using JetPEI (PolyplusTransfection, France) in 35mm plates and harvested at the indicated time points after transfection. The Sf9 cell coinfections were done as described in [27].

### Immunisation and antibody production

The GTRSGSKQSTNPADNYHLA(C) peptide corresponding to human TAF8 amino acids 14–32 was synthesised, coupled to ovalbumin and used for generation of mouse monoclonal antibody (2TAU 2B8) as described in [5]. All the other antibodies used have been previously described in [7], [15], [25], [28], [30], [44].

### FRET measurements

For the fluorescence resonance energy transfer (FRET) measurements a Leica TCS RS microscope was used that was equipped with a 40 mW Ar laser. The images were acquired with 458 and 514 nm laser lines, which were adjusted to 10.5 and 2% maximal laser power, respectively. Images were taken through a 63×1.4-numerical aperture oil immersion objective with 2–4 × zoom. Recording was done with two photomultiplier tubes (PMT1 and PMT2). Tuneable split apertures defined the acquired bandwidth as 468–501 nm for PMT1 and 523–600 nm for PMT2. We recorded images in three configurations: i) excitation at 458 nm, acquisition with PMT1 ( = A); ii) excitation at 458nm, acquisition with PMT2 ( = B), and iii) excitation at 514 nm, acquisition with PMT2 ( = C). Images were acquired of single and double transfected cells in all three configurations. FRET, indicating an interaction between the CFP and YFP fusion proteins was determined from the enhanced emission of the acceptor YFP in the presence of the CFP-tagged partner. The a, b, and c constants indicating spectral bleed thoroughs were determined and averaged on a set of 20 cells with the Leica Confocal Software FRET Application. FRET efficiency, defined as the fraction of emitted acceptor energy originating from Förster resonance energy transfer from the donor was then calculated with the formula: FRET_efficiency_ (%) = [(B-b_*_A-(c-a_*_b)_*_C)/C]_*_100, where (a) is A/C in cells transfected with only the acceptor expression vector; (b) is B/A in cells transfected with only the donor expression vector; and (c) is B/C in cells transfected with only the acceptor expression vector. A, B and C are defined above.

### Preparation of HeLa cell nuclear extract

HeLa cells were grown in suspension culture. 10^11^ cells were harvested by centrifugation and a nuclear extract was prepared according to a modified protocol of Dignam [45]. Briefly, nuclei were prepared by resuspending the pellets in 4 packed cell volume (PCV) of 50 mM Tris-HCl, pH 7.9; 1 mM EDTA; 1 mM DTT and proteinase inhibitors and opening the cells with a Dounce-homogenizer. Nuclei were collected by centrifugation and lysed in 4 PCV of 50 mM Tris-HCl, pH 7.9; 25% glycerol; 500 mM NaCl; 0.5 mM EDTA; 1 mM DTT and proteinase inhibitors by powerful strokes. The lysate was centrifuged at 50000 g for 20minutes. The supernatant was filtered and proteins precipitating in 30%(w/v) (NH_4_)_2_SO_4_ were pelleted. They were resuspended in 50 mM Tris-HCl, pH 7.9; 20% glycerol; 100 mM KCl; 5 mM MgCl_2_; 1 mM DTT and dialysed against the same buffer.

### Immunoprecipitation and Western blot analysis

Sf9 cell lysates were made as described in [28]. Proteins of Sf9 cell lysates (from a 75 cm^2^ Falcon flask) were immunoprecipitated (IP) with 100 µl protein G-Sepharose (Pharmacia) and approximately 5 µg of the different antibodies (as indicated). Antibody-protein G Sepharose bound protein complexes were washed three times with IP buffer (25 mM Tris-HCl pH 7.9, 10% (v/v) glycerol, 0.1% NP40, 0.5 mM DTT, 5 mM MgCl_2_) containing 0.5 M KCl and twice with IP buffer containing 100 mM KCl. After washing, proteins were eluted either by an excess of the corresponding epitope peptide or protein-G-antibody-bound proteins were directly boiled in SDS sample buffer and separated by SDS-PAGE. Proteins were then both visualized by staining the gels with Coomassie blue (or silver nitrate) or transferred to nitrocellulose membrane, and probed with the indicated primary antibodies. Chemiluminescence detection was performed according to manufacturer's instructions (Amersham).

### Expression of proteins in bacteria and GST-pull downs

Transformed BL21DE3 cells were grown at 37°C to O.D._600_ = 0,6-0,8. They were induced with 0.1mM IPTG and grown at 25°C for 45 minutes. The pelleted cells were lysed in PBS-1% TritonX-100, 1 mM DTT, and proteinase inhibitors by sonication. Soluble extracts (2 ml made from a 100 ml culture) were incubated with 50–100 µl of pre-swollen glutathione sepharose (Pharmacia) beads. The beads were extensively washed with lysis buffer (see above) and then further incubated with Sf9 whole cell extracts, in which the indicated interaction partners were overexpressed, for 2h at 4°C. Beads were again extensively washed 3 times with IP buffer containing 500 mM KCl and then twice with IP buffer containing 100 mM KCl. Bead-bound proteins (10 µl) were directly boiled in SDS sample buffer and separated by SDS-PAGE. Proteins were then detected by western blot analysis (as above).

### Mass spectrometry

TFTC subunits were separated on a 10% SDS-PAGE gel. Protein bands were visualized by Coomassie G250 (Biorad) staining, excised and in gel-digested with trypsin [46]. Peptides were either microsequenced [47] or analysed by MALDI mass spectrometry. For MALDI analysis peptide extracts (0.5 µl) were mixed with an equal volume of saturated alpha-cyano-4 hydroxycinnamic acid (LaserBio Labs) dissolved in 50% acetonitrile and applied to the target. Mass measurements were carried out on a Bruker Reflex IV MALDI-TOF spectrometer in the positive-ion reflector mode. The acquisition mass range was 800–3000 Da with low mass gate set at 700 Da. Internal calibration was performed using autolytic trypsin peptides (MH^+^ with *m/z* = 842.51, 2211.11 and 2807.47). Mono-isotopic peptide masses were assigned manually using the Bruker X-TOF software. Database searches were performed using Profound program (http://prowl.rockefeller.edu/) with the following parameters: database NCBI, proteins of human origin, molecular mass between 20–100 kDa, trypsin digestion with one missed cleavage allowed, cysteines modified by carbamidomethylation, methionine oxidation and mass tolerance of 75 ppm.

### Gel filtration chromatography

HeLa nuclear extract (∼300 µg total protein in 50 µl) or immunupurified and eluted TAF10-containing complexes were injected on a Superose 6 PC3.2/30 size exclusion chromatography column using the SMART FPLC system (Pharmacia) and separated at a flow rate of 10 µl/min in buffer GF (25 mM Tris-HCl pH 7.9, 10% (v/v) glycerol, 1 mM DTT, 5 mM MgCl_2_, 300 mM NaCl). When immunopurified complexes were separated the GF buffer contained also 50 µg/ml insulin. Forty fractions (50 µl) were collected and analysed by western blot.

## Supporting Information

Figure S1 Pair wise BestFit alignment between the C-terminal half of yeast Spt7p and the whole length human SPT7-Like protein (see O94864 and AAG47636). The amino acid positions in the different sequences are labelled on the left and on the right. The peptides identified in hSPT7L by MALDI TOFF mass spectrometry are shown in bold and with capital letters. When two peptides follow in a row the trypsin-cutting site is shown by a black triangle. The putative histone fold domain (HFD) in both proteins is over layered (according to [1]). Percent similarity between the two proteins is: 45.98% and percent identity is 22.86%. 1) Gangloff YG, Sanders SL, Romier C, Kirschner D, Weil PA, et al. (2001) Histone folds mediate selective heterodimerization of yeast TAF(II)25 with TFIID components yTAF(II)47 and yTAF(II)65 and with SAGA component ySPT7. Mol Cell Biol 21: 1841–1853
(0.02 MB DOC)
