# Supplementary material for: Identification of a Small TAF Complex and Its Role in the Assembly of TAF-Containing Complexes
Source: PLoS One. 2007 Mar 21;2(3):e316. doi: 10.1371/journal.pone.0000316 (PMC1820849; doi:10.1371/journal.pone.0000316)
Supplement: Figure S1 — Pair wise BestFit alignment between the C-terminal half of yeast Spt7p and the whole length human SPT7-Like protein (see O94864 and AAG47636). The amino acid positions in the different sequences are labelled on the left and on the right. The peptides identified in hSPT7L by MALDI TOFF mass spectrometry are shown in bold and with capital letters. When two peptides follow in a row the trypsin-cutting site is shown by a black triangle. The putative histone fold domain (HFD) in both proteins is over layered (according to [1]). Percent similarity between the two proteins is: 45.98% and percent identity is 22.86%. 1) Gangloff YG, Sanders SL, Romier C, Kirschner D, Weil PA, et al. (2001) Histone folds mediate selective heterodimerization of yeast TAF(II)25 with TFIID components yTAF(II)47 and yTAF(II)65 and with SAGA component ySPT7. Mol Cell Biol 21: 1841–1853 (0.02 MB DOC) [file pone.0000316.s001.doc]

. . . . .

ySpt7 743 kvraeiclkrteyfkngklnsdseaflknpqrmkrfdqlfleykeqkale 792

: ::||.: ..:. |..| .:|. :| : :|

hSPT7L 2 lr**YWGEIPI**......**SSSQTNRSSFDLLP**...............**R**efrlve 31

. . . . .

793 syrqkieqnsimkngfgtvlkqedddqlqfhndhslngneafekqpndie 842

. ..:.| | | .|:|. ..:. .:. :. ::

32 vhdpplhqpsankpkpptmldipsep.............csltihtiqli 68

. . . . .

843 lddtrflqeydisnaipdivyegvntktldkmedasvdrmlqnginkqsr 892

.: |: . .....| : |||.|.. :.:....... |.:::

69 qhnrrlr**NLITAQAQNQQQTEGVK**teeseplpscpgspplpddl..... 113

. . . . .

893 flankdlgltpkmnqnitliqqirhichkislirmlqsplsaqnsrsnpn 942

|.|..| |.| .: |||| .. .:.|

114 ......lpldck.**NPNAPF..QIRHSDPESDFYR**g............... 139

. . . . .

943 aflnnhiynytiiddsldidpvsqlpthdyknnreliwkfmhkniskvam 992

. :||.:|. |.: :.::...:..:

140 .................kgepvtelswhsc.......rqllyqavatila 165

**HFD**

. . . . .

993 angfetahpsainmlteiagdylsnliktlklhhetns.lnrgtnvemlq 1041

.||:.|:.|.:: ||::| :| .:.| |:: : :. |.... .::::

166 hagfdcanesvletltdvaheyclkftkllrfavdrearlgqtpfpdvme 215

. . . . .

1042 ttllenginrpddlfsyvesefgkktkklqdikqklesflrallrptlqe 1091

.: | ||... .| .: : : . . : :|...|.. . ::.|

216 qvfhevgigsvlslqkfwqhrikdyhsymlqiskqlseeyerivnp.... 261

. . . . .

1092 lsernfedesqsfftgdfaseltgedffgfrelglekefgvlsssvplql 1141

|:. ||... :.:: .|::| |...| :|| ::: . |:|:.:

262 ..ekatedakpvkikeepvsdit....fpvse.eleadlasgdqslpmgv 304

. . . . .

1142 lttqfqtvdgetkvqakkiqpeesdsivykkitkgmldagsfwntllpll 1191

|..| : ..:: .|:|. |:.|.. | :|:.:||

305 lgaqserfpsnleveas...pqassaev.........nasplwnl..... 337

. . . . .

1192 qkdyerskayiakqskssandktsmtstednsfalleedqfvskktatka 1241

|.: .:...|.::..|

338 ........ahvkmepqeseegnvs.......................... 353

. . . . .

1242 rlpptgkisttykkkpiasafilpeedlendvkadptttvnakvgaendg 1291

..: |:.|| .:| ..:.. . ::..:

354 .......................ghgvlgsdvfeepmsgmseagipqspd 380

. . . .

1292 dsslflrtpqpldpldmddafddtnmgsnssfsl..slprlnq 1332

|| |.. ||:|. || | | :||

381 ds....................dssygshstdslmgsspvfnqrckkrmrki 412

**Supplemental Figure 1**. Pair wise BestFit alignment between the C-terminal half of yeast Spt7p and the whole length human SPT7-Like protein (see O94864 and AAG47636). The amino acid positions in the different sequences are labelled on the left and on the right. The peptides identified in hSPT7L by MALDI TOFF mass spectrometry are shown in bold and with capital letters. When two peptides follow in a row the trypsin-cutting site is shown by a black triangle. The putative histone fold domain (HFD) in both proteins is over layered (according to [1]). Percent similarity between the two proteins is: 45.98% and percent identity is 22.86%.

1) Gangloff YG, Sanders SL, Romier C, Kirschner D, Weil PA, et al. (2001) Histone folds mediate selective heterodimerization of yeast TAF(II)25 with TFIID components yTAF(II)47 and yTAF(II)65 and with SAGA component ySPT7. Mol Cell Biol 21: 1841-1853
